# Loss of CCM3 impairs DLL4-Notch signalling: implication in endothelial angiogenesis and in inherited cerebral cavernous malformations

**DOI:** 10.1111/jcmm.12022

**Published:** 2013-02-07

**Authors:** Chao You, Ibrahim Erol Sandalcioglu, Philipp Dammann, Ute Felbor, Ulrich Sure, Yuan Zhu

**Affiliations:** a Department of Neurosurgery, University of Duisburg-EssenEssen, Germany; b Department of Neurosurgery, Tongji Hospital, Tongji Medical College, Huazhong University of Science and TechnologyWuhan, China; c Institute of Human Genetics, University Medicine Greifswald and Interfaculty Institute of Genetics and Functional Genomics, University of GreifswaldGreifswald, Germany

**Keywords:** CCM3/PDCD10, DLL4-Notch signalling, angiogenesis, endothelium, cerebral cavernous malformation

## Abstract

CCM3, a product of the *cerebral cavernous malformation 3* or *programmed cell death 10* gene *(CCM3/PDCD10)*, is broadly expressed throughout development in both vertebrates and invertebrates. Increasing evidence indicates a crucial role of CCM3 in vascular development and in regulation of angiogenesis and apoptosis. Furthermore, loss of CCM3 causes inherited (familial) cerebral cavernous malformation (CCM), a common brain vascular anomaly involving aberrant angiogenesis. This study focused on signalling pathways underlying the angiogenic functions of CCM3. Silencing *CCM3* by siRNA stimulated endothelial proliferation, migration and sprouting accompanied by significant downregulation of the core components of Notch signalling including *DLL4, Notch4, HEY2* and *HES1* and by activation of VEGF and Erk pathways. Treatment with recombinant DLL4 (rhDLL4) restored DLL4 expression and reversed *CCM3*-silence-mediated impairment of Notch signalling and reduced the ratio of *VEGF-R2* to *VEGF-R1* expression. Importantly, restoration of DLL4-Notch signalling entirely rescued the hyper-angiogenic phenotype induced by *CCM3* silence. A concomitant loss of *CCM3* and the core components of DLL4-Notch signalling were also demonstrated in *CCM3*-deficient endothelial cells derived from human CCM lesions (CCMEC) and in a *CCM3* germline mutation carrier. This study defined DLL4 as a key downstream target of CCM3 in endothelial cells. CCM3/DLL4-Notch pathway serves as an important signalling for endothelial angiogenesis and is potentially implicated in the pathomechanism of human CCMs.

## Introduction

Cerebral cavernous malformation (CCM), classified as sporadic and familial (inherited) forms, is one of the most common cerebral vascular anomalies involving aberrant angiogenesis. Loss-of-function mutations in one of the three *CCM* genes, namely *CCM1/KRIT1, CCM2/MGC4607* and *CCM3/PDCD10,* predispose to CCM and are associated with up to 90% of familial CCM [1]. Of these three genes, *CCM3* is the most recently discovered and this gene is highly conserved in both vertebrates and invertebrates [2]. *CCM3* is also referred as *PDCD10 (programmed cell death 10)* owing to the up-regulation of its mRNA expression by apoptotic stimuli *in vitro*. It has been known that CCM3 is broadly expressed, including in neuronal and endothelial cells [3, 4]. Despite its neuronal expression pattern and the discovery of vascular pathology after targeted deletion of *Ccm3* in murine neuroglia [5], intensive studies have been carried out for identifying endothelial functions of these *CCM* genes. Indeed, it has recently been recognized that human *CCM3* mutation carriers display an earlier symptom onset already before 15 years of age and a higher risk for cerebral haemorrhage during childhood [6, 7]. This genotype–phenotype association has raised high interest for researchers to address the detailed functions and signalling pathways underlying CCM3. We have recently shown that silencing *CCM3* stimulated angiogenesis with the most prominent effect on endothelial sprouting and tube branching [8]. In contrast, overexpression of *CCM3* inhibited endothelial proliferation, migration and tube formation [9]. Endothelia-specific deletion of *Ccm3* in mice led to lethal embryos associated with angiogenesis defects and with disruption of vascular integrity [10, 11]. Cardiac and cranial vascular defects were also found in *Ccm3*-deficient zebrafish [12, 13]. Furthermore, endothelial loss of heterozygosity for *Ccm3* in postnatal mice resulted in vascular lesions that resemble typical human CCM [10]. These data indicate that *CCM3-*deficiency impairs vascular development/maturation, activates angiogenesis and causes CCM-like lesion.

The presence of CCM3 in the protein complex of CCM1-CCM2 has been shown, suggesting common or related pathways for these three proteins [13, 14]. Additional distinct signalling pathways underlying the angiogenic functions of CCM3 have also been demonstrated [10–12, 15, 16]. CCM3 binds the GCK-III family of sterile 20-like serine/threonine kinases STK24, STK25 and MST4 [13, 15, 17–19]. Of note, this signalling context is essential for vascular development in zebrafish [13, 16] and for cell survival after oxidative stress [20]. CCM3 has also been shown to bind paxillin [21] and membrane protein VEGF-R2 [11] and to interact with heart of glass homolog1 (HEG1) [22], thereby, respectively, regulating cell adhesion, angiogenesis and vascular integrity.

Notch signalling is an evolutionarily conserved pathway that is indispensable for cell-fate determination and vascular development [23]. In mammals, four Notch receptors (Notch1-4) and five ligands (DLL1, DLL3-4 and Jagged1-2) have been identified [24]. DLL4 is the most recently identified Notch ligand and also the sole Notch ligand expressed predominantly by the vascular endothelium. *In vitro* studies have shown that DLL4 is readily able to signal through each of the four human Notch receptors [24]. Regulation of endothelial sprouting is one of the most predominant roles of DLL4-Notch signalling. Activation of DLL4-Notch signalling inhibits excessive tip-cell formation and sprouting in cultured cells, in animal embryos and during tumour angiogenesis [25]. Conversely, loss of DLL4 expression causes dramatic increase in sprouting and branching as a result of excessive tip-cell formation and endothelial proliferation [26]. Therefore, DLL4-Notch is defined as a negative pathway regulating angiogenesis. Indeed, DLL4-Notch pathway has also been implicated in controlling post-angiogenic blood vessel remodelling and to modulate vasoconstriction and blood flow [27]. Interestingly, down-regulation of *DLL1* and *Notch4* was detected in *Ccm1*-deficient mouse embryos [1, 28]. Overexpression of CCM1 inhibited sprouting angiogenesis by activating Notch signalling [29].

We have recently studied the angiogenic properties of *CCM1*-*3* genes in different types of endothelial cells and noted that *CCM3* silence induced the most potent angiogenic phenotype in comparison with that induced by silencing *CCM1* or *CCM2* [8, 30]. The distinct and massive angiogenic phenotype caused by *CCM3* silence is in accordance with the clinical observation that CCM3 patients showed the most aggressive presentations according to genotype–phenotype analysis [8, 31]. It is noteworthy that the hyper-angiogenic phenotype, including increase in proliferation, migration and particularly massive sprouting and tube brunching, mediated by *CCM3* silence was entirely mimetic that induced by inhibition of Dll4-Notch signalling [26, 31]. We have therefore suggested in this study that *CCM3* deficiency stimulated endothelial angiogenesis through impairing DLL4-Notch signalling. To address this hypothesis, we studied whether and which core components as well as the potential downstream pathways underlying DLL4-Notch signalling were affected by *CCM3* silencing. Furthermore, we investigated whether modulation of endothelial DLL4-Notch signalling was able to rescue the angiogenic phenotype and reverse the altered cellular signalling caused by *CCM3* silencing. Finally, the pathological relevance of *CCM3* deficiency and DLL4-Notch signalling was further strengthened by the studies using CCM-derived endothelial cells (CCMEC) as well as human CCM specimens.

## Materials and methods

### Cell culture and CCM gene silencing

Human umbilical vein endothelial cells (HUVECs, Promocell GmbH, Heidelberg, Germany) and human brain microvessel endothelial cells (HBMECs, Provitro GmbH) were cultured according to the manufacture's protocols. Human CCM lesion-derived endothelial cells (CCMECs) were prepared from the surgical specimens of sporadic CCM and cultured as described previously [8, 31]. The diagnosis of sporadic CCM was based on the specific characteristics of magnetic resonance imaging (MRI), multiplicity of the lesion, familial history and genetic background. *CCM3* silence was achieved by transfection of the endothelial cells with specific siRNA targeting *CCM3* genes (siCCM3; Applied Biosystems/Ambion, Darmstadt, Germany) using established protocols [8, 30]. The Negative control siRNA (Neg. C, Applied Biosystems/Ambion) is comprised of a 19 bp scrambled sequence with 3′dT overhands. The sequence has no significant homology to any known gene sequence from human. The efficiency of *CCM3* silencing was confirmed by real-time reverse transcription polymerase chain reaction (RT^2^-PCR) in all experiments when siRNA transfection was concerned.

### Human brain specimens

The diagnosis of CCMs was based on specific characteristics of magnetic resonance imaging, histopathological criteria and genetic background. Operative specimens from six sporadic CCMs including three female and three male with mean age of 42 ± 12 years were used as control. The familial CCM case was a 9-year-old boy and had been shown to carry a frameshift mutation in exon seven of the *CCM3* gene (c.350_351insT, p.D118RfsX2) [14, 32]. All participants enrolled in the study provided informed consent. The experimental protocol was approved by the local ethics committee.

### Real-time RT-PCR (RT^2^-PCR)

The RNA extraction, cDNA synthesis, preparation of PCR reaction mixture and the PCR settings were carried out as described previously [30]. Primer sequences and annealing temperature for PCR were shown in Table 1. GAPDH (Glyceraldehyde 3-phosphate dehydrogenase) was stably detected in cell and tissues samples under the conditions of this study and was thus selected as reference gene. Relative mRNA expression of the target gene (fold of change) for each sample was quantified using the cycle threshold (Ct) approach, normalized to the reference gene.

**Table 1:** Primer sequences and annealing temperatures for real-time RT-PCR

Primer		Sequence	T* (°C)
*CCM1*	For.	TGA AGG AAG CAA TTA ACA AAC CA	60
	Rev.	GAG AGA CGC ATT CCT TCC AT	
*CCM2*	For.	CCC TGT CGG AGA GTG CAG	59
	Rev.	AGC AGA CAG CAA AGC TCC TC	
*CCM3*	For.	TGG CAG CTG ATG ATG TAG AAG	58
	Rev.	TCG TGC CTT TTC GTT TAG GT	
*DLL4*	For.	GCG GGG TAC CTT CTC GCTCAT CATC	60
	Rev.	GCC TCC CCA GCC CTC ATC ACA AGT A	
*Jagged1*	For.	TCG CTG TAT CTG TCC ACC TG	60
	Rev.	AGT CAC TGG CAC GGT TGT AG	
*Notch1*	For.	CAG GCA ATC CGA GGA CTA TG	60
	Rev.	CAG GCG TGT TGT TCT CAC AG	
*Notch4*	For.	TCC TGG GGC CCG GGC TGA AGA AAA G	58
	Rev.	ACG CCG GAT GAG CTG GAG GAC GAG A	
*HEY2*	For.	GTA CCA TCC AGC AGT GCA TC	60
	Rev.	AGA GAA TTC AGT CAG GGC ATT T	
*HES1*	For.	AGT GAA GCA CCT CCG GAA C	60
	Rev.	CGT TCA TGC ACT CGC TGA	
*VEGF*	For.	GAA GTG GTG AAG TTC ATG GAT GT	60
	Rev.	TGG AAG ATG TCC ACC AGG GTC	
*VEGF-R1*	For.	AGC TCC GGC TTT CAG GAA GAT A	58
	Rev.	GAC AGG AAC TCC ATG CCT CTG	
*VEGF-R2*	For.	CTC TTG GCC GTG GTG CCT TTG	58
	Rev.	GTG TGT TGC TCC TTC TTT CAA C	
*GAPDH*	For.	AGC CAC ATC GCT CAG ACA	58
	Rev.	GCC CAA TAC GAC CAA ATC C	

* T: Annealing temperature; for, forward; rev, reverse.

### Western blotting

Total protein extraction and the electrophoresis were performed as described previously [33]. The blots were incubated at 4°C overnight with the following first antibodies: DLL4, p-Erk1/2, p-Akt and GAPDH (each 1:1000 dilution, Cell Signaling, Frankfurt am Main, Germany); Notch4 [1:200, specifically detect the cleaved domain (active form) of the protein, Santa-Cruz Technology, Heidelberg, Germany], VEGF (1:1000; Abcam, Cambridge, UK), Hey1 (1:200, Abcam), CCM3 (1:400; Atlas Antibodies, Stockholm, Sweden) and actin (1:1000; Sigma-Aldrich, Seelze, Germany). After incubation with the corresponding HRP-conjugated secondary antibody, the signal was produced by enhanced chemiluminescence detection reagents. To semi-quantify the blot, integrate optical density (IOD) of the individual blot was measured using Image J software. The IOD ratio of the target protein to the housekeeping protein GAPDH was calculated and the relative expression of the target protein was normalized to the percentage of the control.

### Immunofluorescence

Cells were fixed with methanol at −20°C for 20 min. followed by immunofluorescent staining as previously described [31]. For paraffin-embedded brain sections, immunofluorescent staining of CCM3, DLL4 and vWF was simultaneously performed on the adjacent sections. After deparaffinization, the non-specific binding was blocked by incubation of sections with the blocking buffer [31]. The slices were then incubated with respective rabbit anti-human CCM3 (1:65, Atlas Antibodies) or rabbit anti-human DLL4 (1:100, AbD Serotec, Düsseldorf, Germany) or rabbit anti-human vWF (1:200, Dako, Hamburg, Germany) at 4°C overnight. Negative control sections were incubated with non-immune rabbit IgG at the concentrations identical to the primary antibodies. The immunoreactivity of CCM3 was detected using biotinylated goat anti-rabbit IgG followed by incubation with fluorescein avidin D. The signal for the staining of DLL4 and vWF was induced by the incubation of the slices with a texas-red conjugated anti-rabbit IgG. Counterstaining was performed with Hoechst-33258. The sections were analysed by using a fluorescence microscope (Olympus BX51, Hamburg, Germany). Images were acquired using an Olympus DP 70 camera driven by Olympus software.

### Proliferation and migration assay

The cell proliferation was detected by WST-1 assay as described previously [31]. For migration assay, cells were seeded onto culture dishes pre-coated with 0.2% gelatin. When the cells reached 95–100% confluence, a thin stripe of cells was scratched by a sterile 1000-μl pipette tip. Six fields per culture dish (4 dishes/group) were randomly taken for photograph and the number of the migrated cells in each field was blindly counted.

### Endothelial sprouting assay in 3D gel

The sprouting assay was performed according to Nakatsu *et al*. [34] with modifications. Cells were mixed with Cytodex 3 beads in a ratio of 400 cells per bead followed by the incubation overnight. After washing, the beads were resuspended in a medium supplemented with 2.5 mg/ml of fibrinogen and 0.15 units/ml of aprotinin. The mixture was quickly added to the well of a 96-well plate containing 0.625 U/ml of thrombin to form clot. The clots were overlaid with 100 μl medium containing 10 ng/ml of VEGF (R&D Systems, Wiesbaden-Nordenstadt, Germany) and 0.15 units/ml of aprotinin. Sprouting was monitored and photographed under a microscope. Length of sprouts was measured in 20 randomly chosen beads of each group using Leica Application Suite software.

### Recombinant human DLL4 (rhDLL4) treatment

Cell culture plate was pre-coated with 0.2% gelatin containing different concentrations of rhDLL4 (R&D System) as indicated in individual experiments or the same volume of vehicle (0.1% BSA).

### Statistics

Data were presented as mean and standard deviation (means ± SD). Statistical analysis was performed with the WinSTAT program. Differences between multiple groups were analysed using anova followed by the Scheffé test. *P* value less than 0.05 was considered statistically significant.

## Results

### Characterization of the angiogenic phenotype after silencing *CCM3*

**Fig. 1:**
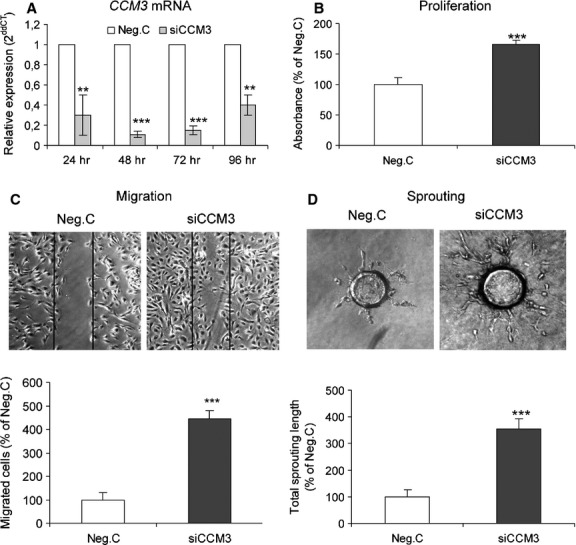
Silencing *CCM3*-activated angiogenesis. (**A**) The time course of *CCM3* silence. HUVECs were transfected with 70 nM of specific *CCM3* siRNA (siCCM3) or with a control siRNA (Neg. C) for different time periods as indicated. The expression of *CCM3* was detected by RT^2^-PCR. Based on this time course, angiogenic assays (**B**–**D**) were accordingly performed 72 hrs after the transfection. (B) Silencing *CCM3-*stimulated endothelial proliferation. HUVEC received 70 nM of siCCM3 or Neg. C. Cell proliferation was detected by using the WST-1 proliferating reagent. (C) Silencing *CCM3* promoted cell migration. The cell migration was recorded 10 hrs after scratching and the number of migrated cells was counted randomly in six fields per culture dish. The data were presented as means of four dishes per group. (D) Silencing *CCM3* resulted in massive angiogenic sprouting in 3D fibrin gel. The total length of sprouts of 20 beads randomly selected from each group was blindly quantified by using Leica Application Suite software. All data were representative of at least three independent experiments. **P < 0.01 and ***P < 0.001, compared with Neg. C.

To ensure a sufficient time window of *CCM3* silence for endothelial behaviour study and for further signalling study, we examined the time course of *CCM3* expression after transfection. RT^2^-PCR revealed a 70%, 90%, 80% and 60% of reduction in *CCM3* expression at 24 hrs (*P* < 0.01), 48 hrs (*P* < 0.001), 72 hrs (*P* < 0.001) and 96 hrs (*P* < 0.01), respectively, after siCCM3 transfection (Fig. 1A). Under this established silencing condition, we then characterized the angiogenic phenotype of endothelial cells. Silencing *CCM3* significantly stimulated all tested angiogenic behaviours including endothelial proliferation (Fig. 1B), migration (Fig. 1C) and sprouting (Fig. 1D).

### Inactivation of DLL4-Notch signalling in *CCM3*-silenced endothelial cells and in a surgical specimen of familial CCM harbouring a *CCM3* germline mutation

**Fig. 2:**
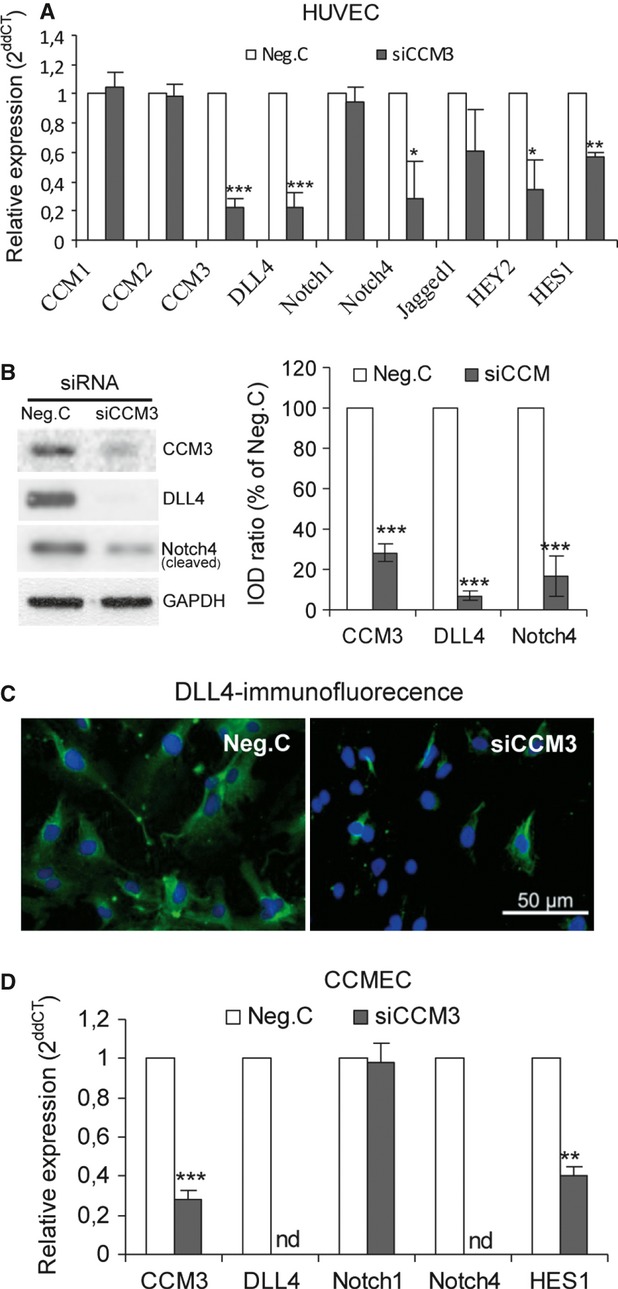
Silencing *CCM3* in cultured endothelial cells down-regulated the core components of DLL4-Notch signalling. Cells were transfected with 70 nM of si*CCM3* or Neg. C. Total RNA or protein was, respectively, extracted 72 hrs after the transfection. (**A**) The mRNA expression of core components of Notch signalling after silencing *CCM3* in HUVEC. si*CCM3* transfection efficiently silenced *CCM3* without influencing the expression of *CCM1* and *CCM2*. Under this experiment conditions, silencing *CCM3* down-regulated the expression of *DLL4, Notch4* and *HEY2* and *HES1*. (**B**) The down-regulation of DLL4 and Notch4 in *CCM3*-silenced HUVEC was confirmed by Western blot. To semi-quantify the blot, integrate optical density (IOD) of the individual blot was measured using ImageJ software. The IOD ratio of the target protein to the housekeeping protein actin was calculated and the relative expression of the target protein was normalized to the percentage of the control (Neg. C). (**C**) Immunofluorescent staining of DLL4. HUVEC was transfected with 70 nM of si*CCM3* or Neg. C for 72 hrs. Immunostaining of DLL4 (green) was performed followed by Hoechst 33258 counterstaining (blue). Scale bar: 50 μm. (**D**) A significant down-regulation of *DLL4, Notch4* and *HES1* was detected in *CCM3*-silenced endothelial cells derived from the lesion of human sporadic CCM (CCMEC). The expression of *DLL4* and *Notch4* was reduced to a non-detectable level (nd) in *CCM3*-silenced CCMEC, whereas the mRNA level of *Notch1* was not altered. All data were representative of at least three independent experiments. *P < 0.05, **P < 0.01 and ***P < 0.001, compared with Neg. C.

To control the efficiency of silencing *CCM3* and to rule out unspecific effect of siCCM3 transfection to *CCM1* and *CCM2*, we examined the expression of *CCM1*, *CCM2* and *CCM3* in parallel in siCCM3 transfected cells. As shown in Figure 2A, the expression of *CCM3*, but not of *CCM1* and *CCM2*, was significantly and specifically silenced by the siCCM3 transfection. Thereafter, the core components of DLL4-Notch signalling were examined in the same RNA extracts as used for *CCM1-3* detection. Interestingly, transfection of siCCM3 caused a five- and threefold decrease in *DLL4* and *Notch4* mRNA expression, respectively, but no influence on *Notch1* expression. Down-regulation of DLL4-Notch signalling was further proven by detection of 60% and 40% decrease in the levels of target genes *HEY2* and *HES1,* respectively, in *CCM3*-silenced HUVEC. Western blot confirmed a more than 10- and 3-fold reduction in the protein levels DLL4 and Notch4 (cleaved/active form) when CCM3 was down-regulated to 20% of the control (Fig. 2B). Immunofluorescent staining confirmed a reduced DLL4 immunoreactivity in *CCM3*-silenced cells (Fig. 2C).

To find out the pathological relevance between CCM3 deficiency and down-regulation of DLL4-Notch signalling, the expression of core components of the DLL4-Notch pathway was further studied in the endothelial cells derived from human CCM lesion (CCMEC) and in surgical specimens of CCMs. As described previously, CCMEC was derived from sporadic CCMs and has been well-established in our laboratory[8, 31]. Silencing *CCM* genes in CCMEC has been used as a useful *in vitro* model for analysing *CCM* gene function and the underlying signalling pathways [8, 31]. As shown in Figure 2D, silencing *CCM3* dramatically down-regulated the expression of *DLL4* and *Notch4* to an undetectable level, and concomitantly accompanied by 60% of reduction in the target gene *HES1*. It was noted that *Notch1* gene expression was not significantly altered by silencing *CCM3* in either CCMEC (Fig. 2D) or HUVEC (Fig. 2A).

**Fig. 3:**
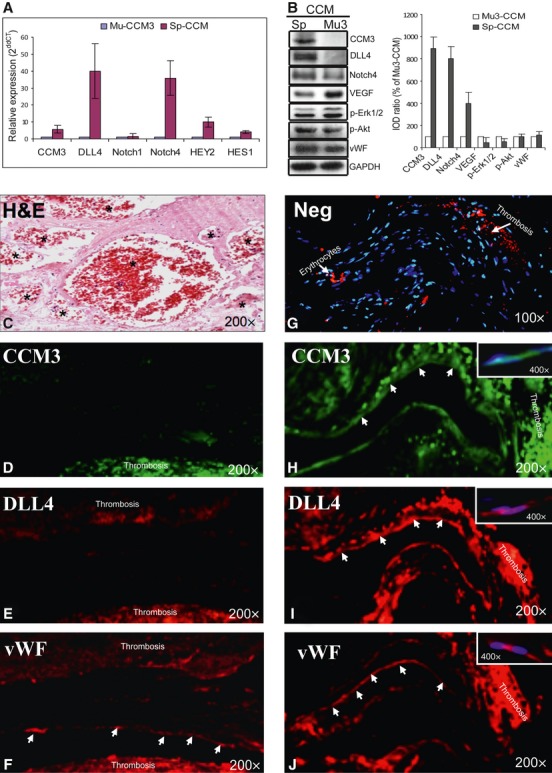
Concomitant down-regulation of CCM3 expression and inactivation of DLL4-Notch signalling in human cavernous tissue derived from a *CCM3* mutation carrier in comparison with the controls (sporadic CCMs). (**A**) Down-regulation of mRNA levels of *CCM3* and the core components of DLL4-Notch signalling in a familial CCM harbouring *CCM3* mutation. Total RNA was extracted from operative specimens of six sporadic CCMs (Sp-CCM) and a familial *CCM3* mutation carrier (Mu-CCM3). The expression of the genes was quantified by RT^2^-PCR. The relative gene expression was calculated when the gene level in familial CCM was normalized as 1. (**B**) Down-regulation of the protein levels of CCM3, DLL4 and cleaved Notch4 accompanied with an up-regulation of VEGF and p-Erk1/2 in human *CCM3*-mutated tissue. The total protein was extracted from the operative specimens of six sporadic CCMs (Sp-CCM) and a familial CCM harbouring *CCM3* mutation (Mu-CCM3). Semi-quantification of the blot was performed by measuring integrate optical density (IOD) of each band followed by calculation of the ratio of IOD from the interested protein to that from the housekeeping protein GAPDH. The data were presented as the percentage of the Mu-CCM3. (**C**) Pathological morphology of CCM revealed by haematoxylin and eosin staining on the section from a sporadic CCM. The lesion consists of immature capillary-like vessels without normal interposed brain parenchyma. These vessels are dilated and often thrombosed (asterisks). (**D**–**J**) Immunofluorescent staining of CCM3, DLL4 and vWF on the sections of human CCM lesions. The immunofluorescent staining was simultaneously performed on the adjacent paraffin sections prepared from a CCM harbouring *CCM3* mutation (Mu3-CCM; **D–F**) and from a sporadic CCM (Sp-CCM; **G–J**). In Mu3-CCM, vWF staining revealed the endothelial layer (arrows) in the thrombosed cavern (**F**), whereas the immunoreactivity of CCM3 (**D**) and DLL4 (**E**) was absent in these endothelial cells. In Sp-CCM, CCM3 (**H**), DLL4 (**I**) and vWF (**J**) were concomitantly detected in the endothelial layer (arrows) of the adjacent sections. Negative control omitting the primary antibody (Neg; **G**) did not show specific staining, and revealed only unspecific signal (red) in thrombosis and in erythrocytes (arrows) which appeared nuclear staining (blue) negative. The original magnification was 200× in main graphs and 400× in white box.

Next, we examined DLL4-Notch signalling in human CCMs. Because of the extremely rare availability, only one human surgical specimen of a *CCM3* germline mutation carrier could be obtained during this study. To draw a more convinced comparison between familial and sporadic CCMs, we used six surgical specimens from sporadic CCM as control. RT^2^-PCR detected around 5-, 38- and 35-fold decrease in *CCM3, DLL4* and *Notch4* expression, respectively, in the *CCM3*-deficient cavernous lesion (Mu-CCM3) in comparison with the human sporadic CCMs (Sp-CCM). A dramatic down-regulation of the target gene *HEY2* and *HES1* was also observed in Mu-CCM3, indicating an impaired DLL4-Notch signalling in familial CCM with germline mutation in *CCM3*. Interestingly, the level of *Notch1* was similarly detected in Mu-CCM3 and in Sp-CCMs (Fig. 3A). These data drawn from CCM patients were entirely consistent with the results obtained from *CCM3*-silenced HUVEC (Fig. 2A) and CCMEC (Fig. 2D).

Western blotting confirmed a significantly lower CCM3 protein level concomitantly with a marked down-regulation of DLL4 and Notch4 in the lesion of the *CCM3* mutation carrier (Mu-CCM3). Semi-quantification of the blot using housekeeping protein GAPDH as the reference protein demonstrated nine-, eight- and fourfold higher levels of CCM3, DLL4 and Notch4, respectively, in the sporadic CCM lesions (Sp-CCM) in comparison with that in Mu3. Conversely, the protein levels of VEGF and p-Erk1/2, but not of p-Akt, were clearly elevated in the tissue derived from the *CCM3*-mutation carrier (Fig. 3B).

It has been known that both sporadic and familial CCMs display undistinguishable histopathological morphology. As revealed by haematoxylin and eosin staining in the section from a sporadic CCM, the lesion typically consists of enriched immature capillary-like vessels often fulfilled with thrombosis (asterisks) without normal interposed brain parenchyma (Fig. 3C). In negative control of immunofluorescent staining omitting the primary antibody (Neg; Fig. 3G), unspecific signal was present in the thrombosis and erythrocytes (arrows), but there was no specific immunoreactivity in the endothelial layer of the covens. To define the association of CCM3 and DLL4 expression in the endothelial layer of CCM covens, immunofluorescent staining of CCM3, DLL4 and vWF was simultaneously performed on adjacent sections from the *CCM3*-mutation carrier (Mu-CCM3; Fig. 3D–F) and from sporadic CCM (Sp-CCM; Fig. 3H–J). Staining of vWF clearly marked the endothelial layer (arrows) in both Mu-CCM3 (Fig. 3F) and Sp-CCM (Fig. 3J). Immunoreactivity of CCM3 (Fig. 3D) and DLL4 (Fig. 3E) was specifically absent in the endothelial layers of the cavernous lesion on the adjacent sections of Mu-CCM3, but was detected in the endothelial layers (marked by arrows and showed at higher magnification in white box) of Sp-CCM (Fig. 3H–J), indicating a concomitant expression pattern of these two proteins in the endothelial cells of the cavernous lesion.

### Recombinant human DLL4 (rhDLL4) recaptured DLL4-Notch signalling and rescued the hyper-angiogenic phenotype of *CCM3*-silenced endothelial cells

**Fig. 4:**
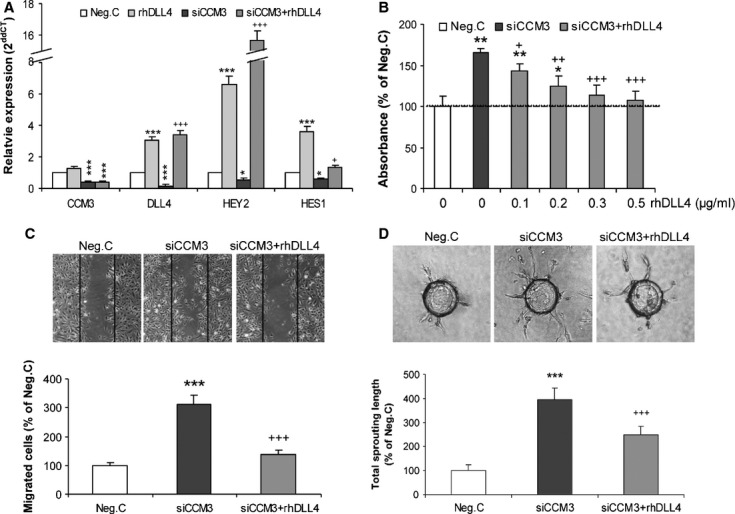
Restoration of DLL4 by treatment with recombinant human DLL4 (rhDLL4) rescued the hyper-angiogenic phenotype of *CCM3*-silenced ECs. (**A**) Treatment with rhDLL4 led to up-regulation of mRNA levels of *DLL4, HEY2* and *HES1* and recaptured *CCM3*-silence-mediated inactivation of Notch signalling. HUVEC received the pre-treatment of 1 μg/ml of rhDLL4 or vehicle (0.1% BSA) for 24 hrs followed by transfection with either siCCM3 (70 nM) or Neg. C. Total RNA was extracted 72 hrs after the transfection for detecting *CCM3* expression and for detecting Notch signalling core components by RT^2^-PCR in parallel. (**B**) rhDLL4 abolished the proliferation effect mediated by *CCM3* silence. After 48 hrs of transfection of siCCM3 or Neg. C, HUVECs were reseeded and incubated for 24 hrs on the culture wells pre-coated with the different concentration of rhDLL4 as indicated. The proliferation assay was performed by using WST-1 reagent. (**C**) rhDLL4 inhibited the migration activity of *CCM3*-silenced endothelial cells. The transfected cells were incubated in culture well pre-coated with 1 μg/ml rhDLL4 or vehicle. Migrated cells were counted in six random fields in each group at 10 hrs after scratching. (**D**) rhDLL4 suppressed the massive angiogenic sprouting caused by *CCM3* silence. The beads formed from the transfected cells were seeded and cultured in 3D fibrin gel immobilized with 1 μg/ml of rhDLL4 or vehicle for 72 hrs. The length of sprouts was measured in 20 randomly selected beads in each group. All data presented in A–D were reproduced in at least three independent experiments. *P < 0.05, ** *P* < 0.001 and *** *P* < 0.001, compared with Neg. C; +*P* < 0.05, ++*P* < 0.01 and +++*P* < 0.001, compared with siCCM3 alone.

Before testing whether restoration of DLL4 expression by rhDLL4 was able to rescue the hyper-angiogenic phenotype of *CCM3*-silenced ECs, we first determined the role of rhDLL4 in activation of DLL4-Notch signalling. As shown in Figure 4A, treatment of rhDLL4 (1 μg/ml) to non-transfected cells resulted in a threefold increase in *DLL4* mRNA expression and a significantly coincident up-regulation of *HEY2* and *HES1* mRNA levels, demonstrating an activation of Notch signalling by rhDLL4. Moreover, pre-treatment of rhDLL4 (1 μg/ml) recaptured siCCM3-induced inhibition of DLL4-Notch signalling as demonstrated by a complete recovery of the expression of *DLL4, HEY2* and *HES1* (Fig. 4A). Of note, rhDLL4 influenced the *CCM3* expression neither in non-transfected nor in transfected endothelial cells.

Under the established rhDLL4 treatment conditions, we were able to further show that pre-treatment of rhDLL4 inhibited activation of proliferation in a dose-dependent manner mediated by siCCM3 silencing. At higher concentrations (0.3 and 0.5 μg/ml), rhDLL4 completely reversed increased proliferation induced by siCCM3 (Fig. 4B). In migration assays, siCCM3 resulted in a threefold higher number of migrated cells in comparison with the control Neg.C (*P* < 0.001), which was completely abolished by rhDLL4 (*P* < 0.001; Fig. 4C). Silencing *CCM3* largely stimulated sprouting as demonstrated by a fourfold increase in the total length of sprouts (*P* < 0.001). This excessive sprouting was also significantly suppressed by rhDLL4 (*P* < 0.001; Fig. 4D).

### RhDLL4 reversed the expression of VEGF-R1, -R2 and p-Erk affected by silencing *CCM3*

**Fig. 5:**
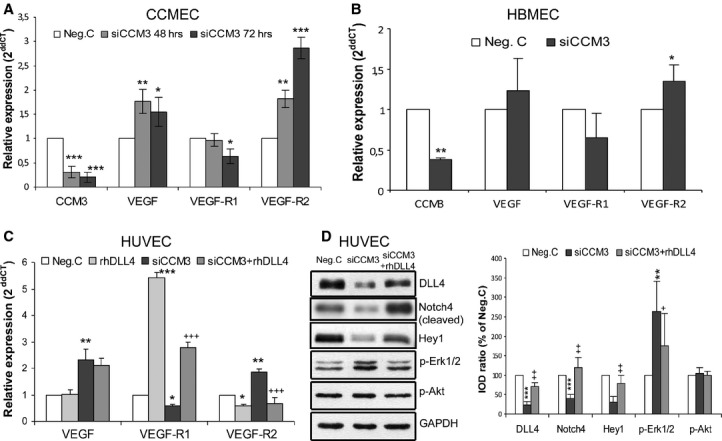
Silencing *CCM3* affected multiple signalling components in cultured endothelial cells, which was reversed by rhDLL4. (**A**) The expression of *VEGF*, *VEGF-R1* and *VEGF-R2* after siCCM3 transfection in CCMEC. CCMECs were transfected with either 70 nM of specific *CCM3* siRNA (siCCM3) or control siRNA (Neg. C). Total RNA was extracted at 48 and 72 hrs after the transfection for RT^2^-PCR. *CCM3* was detected for controlling an efficient silence induced by siRNA transfection. (**B**) The expression of *VEGF*, *VEGF-R1* and *VEGF-R2* after siCCM3 transfection in HBMEC. HBMEC received the transfection with either 70 nM of siCCM3 or Neg. C. Total RNA was extracted 72 hrs after the transfection for RT^2^-PCR. *CCM3* was detected for controlling an efficient silence induced by siRNA transfection. (**C**) The treatment of rhDLL4 reversed the down-regulation of *VEGF-R1* and the up-regulation of *VEGF-R2,* but did not influenced the expression of *VEGF,* in *CCM3*-silenced HUVEC. Cells were transfected with either 70 nM of specific *CCM3* siRNA (siCCM3) or control siRNA (Neg. C) in the presence or the absence of rhDLL4 (1 μg/ml). Total RNA was extracted 72 hrs after the transfection for RT^2^-PCR. (**D**) The treatment with rhDLL4 restored DLL4 protein expression, elevated the levels of cleaved Notch4 and target protein Hey1, and reversed increase in p-Erk1/2 expression mediated by silencing *CCM3*. Total protein was extracted 72 hrs after the transfection in the presence or the absence of rhDLL4 (1 μg/ml). The data presented in A–D were representative of at least three independent experiments. *P < 0.05, **P < 0.001 and ***P < 0.001, compared with Neg. C; +*P* < 0.05, ++*P* < 0.01 and +++*P* < 0.001, compared with siCCM3 alone.

To study the downstream pathway(s) influenced by *CCM3-*silence-mediated inactivation of DLL4-Notch signalling, we examined the expression of *VEGF*, *VEGF-R1* and *VEGF-R2* in different types of cultured endothelial cells. In CCMEC, *CCM3* siRNA transfection resulted in an efficient down-regulation of *CCM3* level to 30% and 20% of the control at 48 hrs (*P* < 0.001) and 72 hrs (*P* < 0.001) respectively. Under these silence conditions, the level of *VEGF* elevated by 76% (*P* < 0.01) and 55% (*P* < 0.01), and more interestingly, the expression of *VEGF-R2* increased by 82% and 187% of control at 48 hrs (*P* < 0.01) and 72 hrs (*P* < 0.001), respectively, after the transfection. In contrast, the *VEGF-R1* mRNA level was moderately down-regulated at 72 hrs after siCCM3 transfection (*P* < 0.05; Fig. 5A). A similar tendency of change in the expression of *VEGF*, and *VEGF-R1* and *VEGF-R2* was observed in *CCM3*-silenced HUVEC (Fig. 5C), whereas *CCM3* silencing in HBMEC resulted in only minor elevation of VEGF-R2 level (Fig. 5B).

Next, we attempted to test whether rhDLL4 can reverse these effects induced by *CCM3* silence. Treatment of rhDLL4 under basal condition caused a dramatic up-regulation of *VEGF-R1,* and meanwhile a down-regulation of *VEGF-R2*. Indeed*,* pre-treatment of rhDLL4 to siCCM3-transfected cells completely reversed the expression of *VEGF-R1* and *VEGF-R2,* but influenced *VEGF* expression neither in basal control nor after siCCM3 transfection (Fig. 5C).

Western blot revealed that treatment with rhDLL4 not only significantly reversed siCCM3-mediated down-regulation of DLL4 but also the protein levels of the Notch4 (active form) and Hey1 (Fig. 5D), indicating that the inhibition of Notch signalling was resulted from the decreased expression of DLL4 after silencing *CCM3*. We also examined the activation status of Erk1/2 and Akt in *CCM3*-silenced ECs. Western blot analyses showed an increased level of p-Erk1/2, but not of p-Akt in *CCM3*-silenced HUVEC. Importantly, the activation of Erk1/2 was reduced after restoration of DLL4 expression by the treatment of rhDLL4 (Fig. 5D).

## Discussion

DLL4-Notch signalling plays a pivotal role in regulating vascularisation, angiogenesis as well as post-angiogenic vessel remodelling and vessel maturation [24, 27]. Compelling evidence indicates that blockade of DLL4-Notch signalling leads to a constitutive angiogenesis [35]. Therefore, DLL4-Notch is defined as a negative pathway regulating angiogenesis. Interestingly, the hyper-angiogenetic phenotype caused by silencing *CCM3* (Fig. 1) completely mimetic that induced by inhibition of DLL4 [31]. This study thus focused on study of DLL4-realted-Notch signalling both in cultured endothelial cells and in human CCM tissues. We provided the evidence that loss of *CCM3*, a gene frequently mutated in familial CCM, significantly down-regulated DLL4 expression and impaired DLL4-Notch signalling thereby activating endothelial angiogenesis. Importantly, a dramatic down-regulation of core components of DLL4-Notch signalling was not only demonstrated in *CCM3*-silenced endothelial cells including HUVEC (Fig. 2A–C) and to a greater extent CCMEC (Fig. 2D) but also in a human lesion known to harbour *CCM3* germline mutation (Mu-CCM3; Fig. 3A and B). The absence of CCM3 immunoreactivity was accompanied by loss of DLL4 expression in the endothelial layer of the cavernous lesion in Mu-CCM3. This suggested a pathological relevance of these two proteins and their potential role in deregulated endothelial function in CCMs (Fig. 3D–F). This proposal was supported by the findings that *CCM3*-silence-induced hyper-angiogenic phenotype was fully rescued by restoration of DLL4 expression *via* treatment with rhDLL4 (Fig. 4A–D), and meanwhile, rhDLL4 treatment entirely reversed *CCM3-*silence-induced down-regulation of core-components DLL4-Notch signalling including DLL4, cleaved (active) Notch4 and target Hey1 (Fig. 5D). Thus, loss of DLL4 seems to be a key downstream event, among others, underlying the pathomechanism of aberrant angiogenesis resulted from *CCM3* deficiency.

Besides well-established role of DLL4 in pro-angiogenesis, increasing evidence indicate a pivotal role of DLL4-Notch signalling in post-angiogenic regulation of vessel remodelling and maturation, a process critical for determining the patterning and density of blood vessels in mature tissues. It has been shown that constitutive or conditional genetic deletion of DLL4 or pharmacologic inhibition of Dll4/Notch signalling inhibited the normal developmental pruning of capillaries and up-regulated the expression of vasodilators but suppressed the expression of vasoconstrictor [27]. Of note, all these phenotypes induced by inhibition of Dll4-Notch signalling are very similar to the typical pathology features of human CCM [36]. It is tempting to speculate that *CCM3*-deficiency-induced down-regulation of DLL4 and the subsequent inactivation of the DLL4-Notch pathway may largely contribute to the severe alteration of the angioarchitecture, which could in part account for the earlier and more severe clinical onset observed in *CCM3* mutation carriers [6, 7]. To our knowledge, this is the first report defining DLL4-Notch as a signalling cascade targeted by *CCM3* deficiency both in endothelial cells as well as in human*-CCM3*-deficient tissue. The results on human operative specimen highlight the pathological relevance of the findings obtained from *in vitro* studies. Meanwhile, we are also aware of the importance to carry out experimental studies on more *CCM3* mutation carriers. However, these studies are limited by the fact that such tissue is extremely rare.

Whitehead *et al*. [28] previously reported that loss of *Ccm1* leads to vasculature defects in embryo associated with loss of *Dll4* and *Notch4*; and they further demonstrated the down-regulation of Dll4 and Notch4 in vessels of human CCM lesion associated with loss of function mutations in *CCM1*. The implication of Notch signalling in the angiogenic function of CCM1 was highlighted by a later study by Wüstehube *et al*. [29]. In light of these previous and the present studies, it is most likely that loss of either CCM1 or CCM3 commonly inhibited DLL4-Notch signalling, which indirectly supported the previous hypothesis that CCM proteins are present in a complex [13, 14]. On the other hand, increasing evidence indicates that individual CCM proteins may target distinct signalling pathways as well [10, 12], suggesting that CCM may be a common tissue manifestation of distinct mechanistic pathways.

Interestingly, CCM3 has been implicated in oxidative stress. CCM3 was up-regulated by different oxidative stimulations, which protected cells from oxidative stress, whereas silencing *CCM3* caused cells death after reactive oxygen species (ROS) exposure [20]. An enhanced ROS levels was also observed in *CCM1*-deficient cells associated with an increase in cell susceptibility to DNA damage [37]. On the other hand, perturbing Notch signalling has been related to the accumulation of ROS [38]. Based on these, it might be interest to elucidate in the future whether loss of either CCM3 or CCM1 induces oxidative stress in endothelial cells through impairment of Notch signalling.

VEGF is a key angiogenesis regulator and VEGF-mediated angiogenesis is primarily linked to VEGF-receptor 2 (VEGF-R2), whereas VEGF-receptor 1 (VEGF-R1) acts as a functional antagonist of VEGF-R2 negatively regulating the angiogenic effects of VEGF [1, 39, 40]. As VEGF-R1 shows a significantly higher affinity to VEGF in comparison with VEGF-R2, a slight change in VEGF-R1 expression is predictive of a marked influence on VEGF-mediated angiogenesis. Regulating the expression of VEGF receptors by Notch signalling has been suggested as an important mechanism to control vascular patterning [25] and to attenuate sprouting activity [1, 39]. Interestingly, CCM3 has been shown to stabilize VEGF-R2 in a mouse model [11]. However, this effect was not observed in another independent mouse model [10] and in a zebrafish model [12]. This disparity may be because of the difference in knockout strategy and animal species.

Accumulating evidence indicates a crucial role of DLL4 in regulating VEGF-R1 and -R2. Deletion of DLL4 reduced the expression of VEGF-R1 but increased expression of VEGF-R2, thereby stimulating endothelial proliferation, migration and sprouting [41, 42]. In contrast, up-regulation of DLL4 expression suppressed angiogenesis because of regressing VEGF-R2 expression but increasing VEGF–R1 level [31, 43, 44]. These data suggest that the level of DLL4 determines endothelial angiogenic activity through regulating the balance of VEGF-R1/VEGF-R2. In consistence with these findings, we showed that rhDLL4 treatment caused a transcriptional down-regulation of *VEGF-R2*, and in a much more extent, an up-regulation of *VEGF-R1* in HUVEC. Furthermore, this study revealed a tendency of increase in the ratio of the mRNA level of *VEGF-R2* to *VEGF-R1* in different types of *CCM3*-silenced endothelial cells and in *CCM3*-deficient human cavernous tissue. Together with the elevated level of *VEGF* found in *CCM3*-silenced endothelial cells, it is feasible to propose that *CCM3* deficiency may activate VEGF signalling. It is noteworthy that rhDLL4 treatment completely reversed the down-regulation of *VEGF-R1* and up-regulation of *VEGF-R2* induced by *CCM3* silencing. These findings highlights that *CCM3* deficiency in endothelial cells alters the balance of the *VEGF-R1* and *VEGF-R2* expression owing to down-regulation of *DLL4* expression, which may potentially disrupt the homeostasis of VEGF signalling thereby leading to hyper-angiogenesis and vassal hyper-permeability.

Erk1/2 is a widely expressed protein kinase that responds to multiple extracellular stimuli. It has been shown that VEGF regulate variety of cell functions including angiogenesis [45, 46], cell permeability [47] and cell viability [48] through modulating Erk activity. Here, we observed an elevated level of p-Erk1/2, concomitantly with the inactivation of DLL4-Notch signalling and with the up-regulation of VEGF in human-*CCM3*-deficient cavernous tissue. Restoration of DLL4 by rhDLL4 treatment not only rescued siCCM3-induced inhibition of DLL4-Notch signalling but also blocked siCCM3-induced Erk1/2 activation, suggesting that Erk1/2 lies downstream of Notch signalling and is likely a target of the VEGF pathway.

**Fig. 6:**
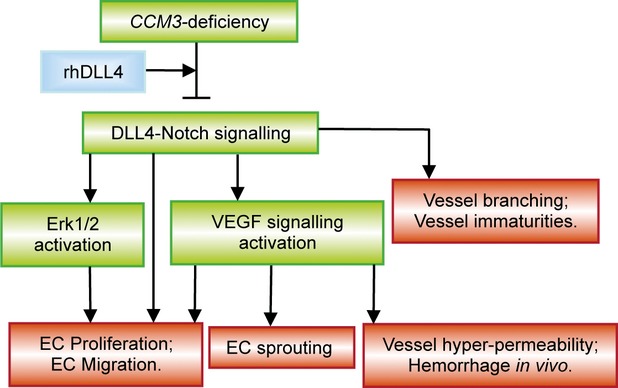
Schematic illustration of the signalling pathways affected by *CCM3* deficiency and possible implication in the pathology of CCM. Loss of CCM3 impaired DLL4-Notch signalling, disrupted the homeostasis of VEGF pathway and activated Erk1/2, which leads to stimulation of endothelial proliferation, migration and sprouting. Treatment with rhDLL4 restored DLL4-Notch signalling, inhibited VEGF and Erk1,2 pathways, and eventually rescued the hyper-angiogenic phenotype induced by *CCM3* silencing, suggesting DLL4 as a key downstream modulator in CCM3 endothelial signalling. In addition to angiogenesis, DLL4-Notch signalling and VEGF signalling are well-defined pathways for regulation of vessel branching/maturation and vessel permeability respectively. We thus suppose that dysregulation of these pathways may be associated with the impairment of these post-angiogenic functions thereby potentially contributing to the enriched immature angioarchitectures with recurrent haemorrhage typically seen in the lesion of human CCMs.

In summary, this study identified an endothelial signalling axle CCM3*/*DLL4-Notch/VEGF/Erk1/2. As illustrated in Figure 6, loss of CCM3 impaired DLL4-Notch signalling, disrupted the homeostasis of VEGF pathway and activated Erk1/2, which was crucially involved in the regulation of endothelial proliferation, migration and sprouting. In addition to angiogenesis, DLL4-Notch and VEGF signallings are well-defined pathways for regulation of vessel maturation and vessel permeability respectively. We thus suppose that dysregulation of these pathways may be potentially associated with the enriched immature angioarchitectures and with recurrent haemorrhage typically seen in the lesion of human CCMs. Treatment with rhDLL4 restored DLL4-Notch signalling, inhibited VEGF and Erk1,2 pathways and eventually rescued the hyper-angiogenic phenotype induced by *CCM3* silencing. These findings highlight DLL4 as a key downstream modulator in CCM3 endothelial signalling and as a potential target for the medication of CCMs.
